# Supplementary material for: Th17-Dependent Nasal Hyperresponsiveness Is Mitigated by Steroid Treatment
Source: Biomolecules. 2022 May 6;12(5):674. doi: 10.3390/biom12050674 (PMC9138412; doi:10.3390/biom12050674)
Supplement: Supplementary file 1 [file biomolecules-12-00674-s001.zip › biomolecules-1649944-supplementary.pdf]

biomolecules

MDPI

Supplementary Material for

Communication

Th17-Dependent Nasal Hyperresponsiveness Is Mitigated by Steroid Treatment

Shusaku Ueda [1,†], Kento Miura [1,†], Hideki Kawasaki [1], Sawako Ogata [1], Norimasa Yamasaki [1], Shuka Miura [1], Akio Mori [2] and Osamu Kaminuma [1,2,*]

[1] Department of Disease Model, Research Institute of Radiation Biology and Medicine, Hiroshima University, Hiroshima 734-8553, Japan; trick_tubatomitu@yahoo.co.jp (S.U.); kmiura@hiroshima-u.ac.jp (K.M.); manumik623@yahoo.co.jp (H.K.); ogata3@hiroshima-u.ac.jp (S.O.); hlnkrcf@hiroshima-u.ac.jp (N.Y.); shu0314@hiroshima-u.ac.jp (S.M.)

[2] Clinical Research Center for Allergy and Rheumatology, National Hospital Organization, Sagamihara National Hospital, Kanagawa 252-0392, Japan; mori-kkr@umin.ac.jp

* Correspondence: okami@hiroshima-u.ac.jp; Tel.: +81-82-257-5819

† These authors contributed equally to this work.

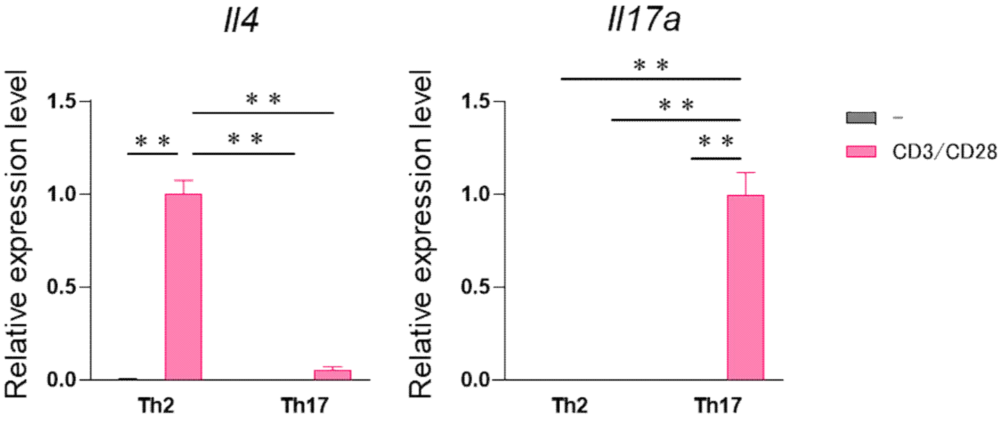

Figure S1. Expression of *Il4* and *Il17a* mRNA in *in vitro*-differentiated Th2 and Th17 cells. The relative expression levels were normalized to *Gapdh* expression as an endogenous reference. Data are displayed as the mean ± standard error of the mean (n = 4). The mean value of Th2 or Th17 activated with anti-CD3/CD28 was set as 1.0 for *Il4* or *Il17a*, respectively. Statistical analyses were conducted by one-way analysis of variance and additional Dunnett's test. $^{**}p < 0.01$ (Dunnett's test).

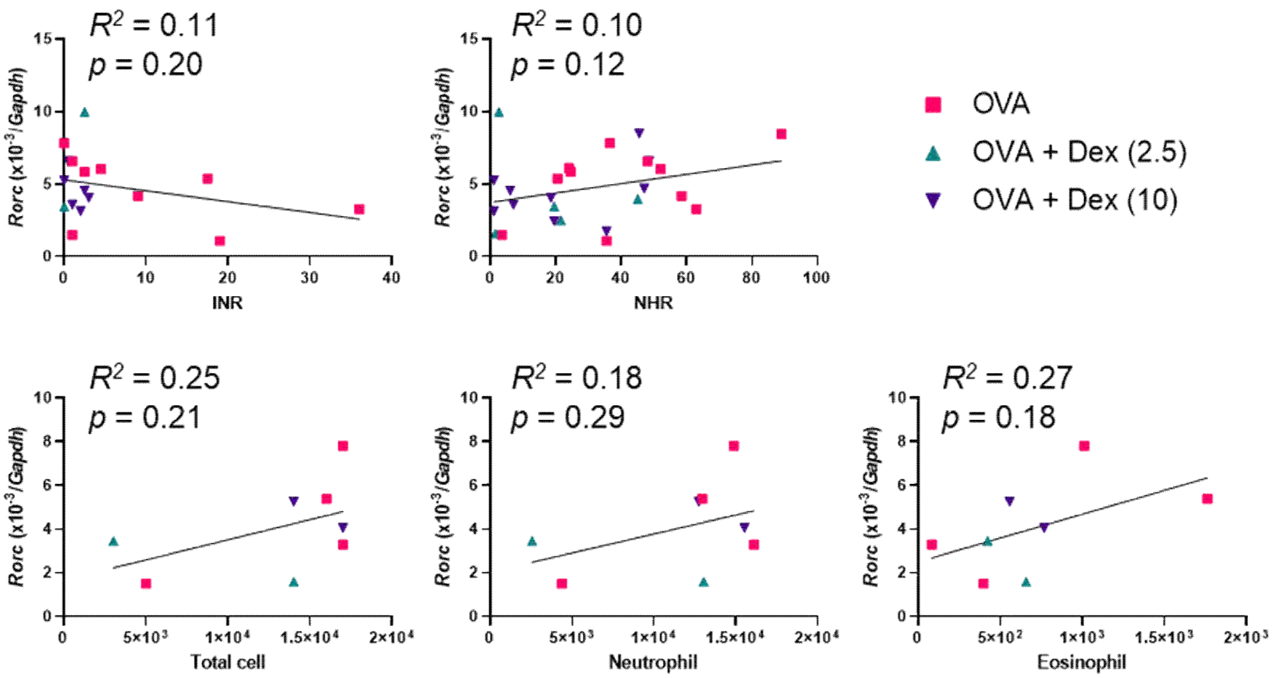

Figure S2. Relationship between nasal *Rorc* expression and inflammatory responses. The correlation of *Rorc* expression in the nasal-associated lymphoid tissue with allergen-induced immediate nasal response (INR), nasal hyperresponsiveness (NHR), and the number of total cell, neutrophils, and eosinophils in the nasal lavage fluids obtained in Th17 cell-transferred and ovalbumin (OVA)-challenged mice was evaluated. The Pearson correlation coefficient (R^2) and *p*-value were also shown. Dex, dexamethasone (2.5 or 10 mg/kg).

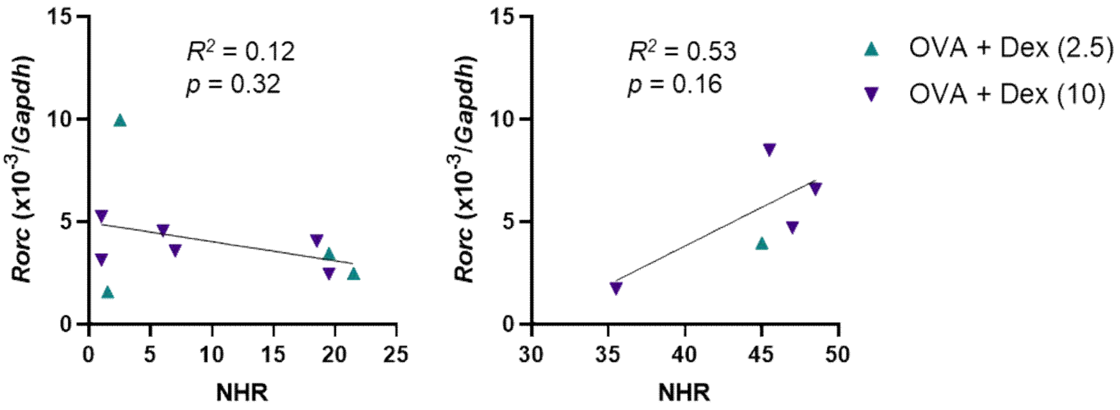

Figure S3. Relationship between nasal *Rorc* expression and nasal hyperresponsiveness (NHR). The correlation between *Rorc* expression in the nasal-associated lymphoid tissue and NHR was separately evaluated for Dex-responder (left panel) and -non-responder (right panel) groups. The Pearson correlation coefficient (R^2) and *p*-value were also shown for individual groups. OVA, ovalbumin; Dex, dexamethasone (2.5 or 10 mg/kg).

In vitro development of allergen-reactive Th2 and Th17 cells

Allergen-reactive Th2 and Th17 cells were developed as previously described [4,8]. In brief, ovalbumin (OVA)-reactive $CD4^+$ T cells were prepared from spleen cells of BALB/c background transgenic mice, DO11.10/$RAG2^{-/-}$, by magnetic cell sorting with an EasySep Mouse $CD4^+$ T Cell Isolation Kit (Veritas, Santa Clara, CA, USA). The cells were co-cultured in the presence of X-ray-irradiated spleen cells in AIM-V medium (Thermo Fisher Scientific, Waltham, MA, USA) with 10% fetal calf serum. At the beginning of culture, we added 0.3 μM OVA323-339 synthetic peptide (Scrum Inc., Tokyo, Japan), 20 U/mL IL-2 (PeproTech, Rocky Hill, NJ, USA), and 10 μg/mL anti-IFN-γ (R4-6A2, eBioscience, San Diego, CA, USA). We further added 10 U/mL recombinant IL-4 (Peprotech) for Th2 development, and 10 ng/mL human IL-1β (PeproTech), 20 ng/mL IL-6 (PeproTech), 10 ng/mL IL-23 (R & D Systems, Minneapolis, MN, USA), 1 ng/mL human TGF-β (Bio-Legend, San Diego, CA, USA), 10 ng/mL TNF-α (PeproTech), and 10 μg/mL anti-IL-4 (Abcam, Cambridge, UK) for Th17 development. Cells were collected following seven-day culture, activated for 6 h by Dynabeads coated with anti-CD3/CD28 (Thermo Fisher Scientific) in AIM-V with 10% fetal calf serum, and processed for the gene expression assessment.

Gene expression assessment

Following the extraction of total RNA from the Th cells, reverse transcription was performed using random primers with SuperScript VILO cDNA Synthesis Kit (Thermo Fisher Scientific), and then quantitative RT-PCR for *Il4* (Mm00439618_m1) and *Il17a* (Mm00439618_m1) was carried out using Taqman gene expression probes (Thermo Fish-er Scientific) on the ABI StepOnePlus™ Real-Time PCR System (Thermo Fisher Scientific). The relative transcript levels were normalized to *Gapdh* (4351309) expression as an endogenous reference using the ΔΔCt method.
